# Editorial: Unlocking the potential of cell therapy: exploring cell types, induction methods, and culture techniques

**DOI:** 10.3389/fcell.2024.1515978

**Published:** 2024-10-30

**Authors:** Viknesvaran Selvarajan, Samuel Ken-En Gan, Yuen Ling Ng

**Affiliations:** ^1^ Bioprocessing Technology Institute, Agency for Science, Technology and Research (A*STAR), Singapore, Singapore; ^2^ Antibody & Product Development (APD) Lab, Wenzhou-Kean University, Wenzhou, Zhejiang, China; ^3^ James Cook University, Singapore, Singapore; ^4^ School of Science & Technology, Singapore University of Social Sciences, Singapore, Singapore; ^5^ Newcastle University in Singapore, Singapore

**Keywords:** cell therapy, chimeric antigen receptor (CAR) T-cells, 3D organoid culture, immunotherapy, mesenchymal stromal cells (MSCs), gene editing (CRISPR-Cas9), bioreactor systems, regenerative medicine

## Introduction

Cell therapy is revolutionizing modern medicine by offering innovative approaches to treating diseases that were previously considered incurable, such as cancer, organ failure, and degenerative conditions. This editorial summarizes key insights and findings from recent research that explores diverse cell types, innovative induction methods, and advanced culture techniques, all of which drive forward the potential of cell-based therapies.

### Cell types in therapy: tailoring treatments for diverse diseases

One of the cornerstones of cell therapy is the ability to select and manipulate specific cell types for therapeutic purposes. Human pluripotent stem cells (PSCs) are valuable in regenerative medicine due to their ability to differentiate into various cell types. Budi et al. demonstrated the advantages of 3D organoid cultures over traditional 2D systems in the generation of functional cholangiocytes, which are critical for treating cholangiopathies. These conditions, often leading to liver failure, currently lack effective treatments beyond transplantation. This advancement in creating mature, functional cells opens the door to cell-based therapies for liver diseases.

Chimeric antigen receptor (CAR) T-cell therapies have shown remarkable success in cancer immunotherapy, particularly in hematologic malignancies like B-cell lymphomas. However, their application in treating acute myeloid leukemia (AML) remains challenging due to difficulties in identifying AML-specific target antigens. Ongoing research, including studies by Guijarro-Albaladejo et al., aims to refine CAR-T technology by improving target specificity and reducing off-target effects. Additionally, Selvarajan et al. highlighted the importance of optimizing bioreactor platforms to expand T-cells, enabling the large-scale production needed for commercial application.

Further expanding the scope of immune cell therapies, CAR-macrophages (CAR-Ms) are emerging as a promising solution for solid tumor treatment. Unlike CAR-T cells, CAR-Ms can thrive within the tumor microenvironment and have shown the potential to persist and effectively combat solid tumors. These findings suggest that CAR-Ms may overcome some limitations of CAR-T cell therapy, which faces challenges in treating solid tumors (Huang et al.).

### Induction methods: enhancing cell functionality for therapeutic applications

In the context of immunotherapy, gene-editing technologies, such as CRISPR, are being employed to enhance CAR-T cell functionality. By refining gene-editing protocols and improving CAR expression, researchers are working to create more specific, potent, and scalable therapies (Guijarro-Albaladejo et al.). The continuous optimization of induction protocols, coupled with advancements in bioprocessing technologies, is crucial for the successful translation of these therapies into clinical practice.

Moreover, the exploration of mesenchymal stromal cells (MSCs) as therapeutic agents for chronic kidney disease (CKD) reveals the importance of delivery methods in maximizing therapeutic efficacy. Gregersen et al. compare systemic and local delivery of preconditioned MSCs, concluding that local administration results in superior outcomes for renal fibrosis by directly targeting the diseased site. Their findings suggest that while MSCs hold great promise in treating fibrosis, the mode of delivery plays a crucial role in determining therapeutic success.

### Culture techniques: scaling up for clinical application

Scaling up the production of therapeutic cells while maintaining their functionality is one of the most pressing challenges in the commercialization of cell therapies. Studies like those by Selvarajan et al. have demonstrated the efficacy of using dynamic bioreactor systems, such as stirred-tank and WAVE bioreactors, to enhance T-cell expansion while preserving cell quality. These bioreactors allow for uniform culture conditions and minimize cell damage, making it feasible to produce large quantities of therapeutic cells.

In regenerative medicine, 3D organoid cultures have emerged as a powerful tool for modeling complex tissue structures and functions. The ability to create organoids that mimic human tissues not only improves cell functionality but also provides a valuable platform for disease modeling and drug testing. These advancements in 3D culture techniques are pushing the boundaries of what is possible in cell therapy, offering more realistic models for studying disease mechanisms and developing new therapies (Budi et al.).

## Challenges and future directions

Despite the significant progress in cell therapy, several challenges remain. Issues such as off-target effects, the scalability of cell production, and the long-term efficacy of therapies continue to limit their widespread clinical adoption. However, innovations in bioprocessing, gene editing, and culture techniques are steadily addressing these obstacles.

The future of cell therapy lies in interdisciplinary collaboration between biologists, engineers, and clinicians. By integrating advanced bioprocessing techniques, optimizing cell delivery methods, and refining immune-modulating therapies, researchers are moving closer to offering accessible, effective, and scalable cell-based treatments. As demonstrated by the research presented in this editorial, the potential for cell therapy to revolutionize healthcare is immense. The next step is ensuring that these therapies reach the patients who need them most.

## Conclusion

Cell therapy holds transformative potential across a wide range of medical fields, from cancer immunotherapy to regenerative medicine. The integration of innovative cell types, advanced induction methods, and scalable culture techniques is paving the way for the next-generation of cell-based therapies. As research in this field continues to evolve, so too does the promise of developing personalized, effective, and safe treatments for a wide array of diseases.

